# Construction of a pathological risk model of occult lymph node metastases for prognostication by semi-automated image analysis of tumor budding in early-stage oral squamous cell carcinoma

**DOI:** 10.18632/oncotarget.15314

**Published:** 2017-02-14

**Authors:** Nicklas Juel Pedersen, David Hebbelstrup Jensen, Giedrius Lelkaitis, Katalin Kiss, Birgitte Charabi, Lena Specht, Christian von Buchwald

**Affiliations:** ^1^ Department of Otorhinolaryngology, Head and Neck Surgery and Audiology, Copenhagen University Hospital, Rigshospitalet, Copenhagen, Denmark; ^2^ Department of Pathology, Copenhagen University Hospital, Rigshospitalet, Copenhagen, Denmark; ^3^ Department of Oncology, Copenhagen University Hospital, Rigshospitalet, Copenhagen, Denmark

**Keywords:** oral squamous cell carcinoma, digital pathology, tumor budding, REMARK guidelines

## Abstract

It is challenging to identify at diagnosis those patients with early oral squamous cell carcinoma (OSCC), who have a poor prognosis and those that have a high risk of harboring occult lymph node metastases. The aim of this study was to develop a standardized and objective digital scoring method to evaluate the predictive value of tumor budding. We developed a semi-automated image-analysis algorithm, Digital Tumor Bud Count (DTBC), to evaluate tumor budding. The algorithm was tested in 222 consecutive patients with early-stage OSCC and major endpoints were overall (OS) and progression free survival (PFS). We subsequently constructed and cross-validated a binary logistic regression model and evaluated its clinical utility by decision curve analysis. A high DTBC was an independent predictor of both poor OS and PFS in a multivariate Cox regression model. The logistic regression model was able to identify patients with occult lymph node metastases with an area under the curve (AUC) of 0.83 (95% CI: 0.78–0.89, *P* <0.001) and a 10-fold cross-validated AUC of 0.79. Compared to other known histopathological risk factors, the DTBC had a higher diagnostic accuracy. The proposed, novel risk model could be used as a guide to identify patients who would benefit from an up-front neck dissection.

## INTRODUCTION

The world-wide incidence of oral squamous cell carcinoma (OSCC) is estimated to be greater than 300,000 new cases a year [1]. OSCC survival and treatment depend largely upon the clinical tumor-node-metastasis (TNM) classification, and 5-year survival in stage I–II OSCC is approximately 80% [2, 3]. Up to 30% of OSCC patients with a clinical N0 (cN0) neck can be demonstrated to harbor occult lymph node metastases when performing a sentinel lymph node biopsy (SNB). The discovery of a single micro- or macroscopic lymph node metastasis confers a poorer prognosis [3, 4]. Identifying which patients with early-stage OSCC who have a high risk of harboring occult lymph metastases is of great clinical importance, since these patients would benefit from therapeutic neck dissection [5].

Several tumor features have been proposed to aid in predicting which patients have occult lymph node metastases at diagnosis, such as gene expression signatures [6–8], tumor depth [3, 9–11] and “worst pattern of invasion” [12]. However, none of these has been implemented consistently in routine clinical practice, some because of a lack of validation [7, 8] and/or a low cost-benefit ratio [6], and others because the complex and time-consuming scoring systems [12] are difficult to implement in a busy pathology department.

The term “tumor budding” was coined by Hase *et al* over 20 years ago, and it is currently defined as isolated clusters of up to five tumor cells [13, 14]. A high tumor bud count at the invasive front have been linked to a poorer prognosis and to a high risk of metastases in several cancer types, including OSCC [15–20]. Despite efforts to standardize and simplify the scoring of tumor buds, scoring remains time consuming, and a certain degree of subjective interpretation remains that results in relatively high inter- and intraobserver variability. Our research group has previously shown the predictive potential of evaluating tumor budding digitally in a mixed group of patients with OSCC who were primarily in an advanced stage [17]. We therefore propose the use of a quantitative, semi-automatic digital image analysis algorithm that would standardize the evaluation of tumor buds in clinical T1-T2N0M0 OSCC. We call this measure of tumor dissociation the Digital Tumor Bud Count (DTBC).

The purpose of this study was thus to evaluate the performance of the DTBC in predicting prognosis and to determine whether the DTBC would be clinically useful in identifying patients with occult lymph node metastases. A clinically useful, reliable, pathological scoring system to identify OSCC patients who are most likely to have poor survival therefore has great clinical importance and applicability.

## RESULTS

### Clinical and pathological characteristics

Table 1 summarizes the clinicopathological characteristics of the included subjects as obtained from surgery and patients hospital records. The median age of the included patients was 64 years (range: 30–95), and the median length of follow-up for those alive at last follow-up was 3.0 years (range: 0.79–7.6 years). The majority of tumors were clinical T1 (cT1) (65%), and 34% had regional disease (Table 1).

**Table 1:** Clinicopathological characteristics

	No.	%
Gender (Male)	126	57
Tumor site		
Floor of the mouth	103	47
Oral tongue	94	42
Other subsites^a^	25	11
UICC Stage^b^		
I	111	50
II	48	22
III	44	20
IVa	19	8
Tumor invasive depth		
<4mm	114	52
>4mm	105	48
Differentiation grade		
High	57	15
Moderate	128	59
Poor	32	26
Tumor invasive front		
Cohesive	83	40
Non-cohesive	123	60
Perineural invasion		
Yes	61	29
No	150	71
Metastases^c^		
N-	147	66
N+	75	34
Smoking^d^		
High	144	78
Low	40	22

a: This category includes other oral subsites such as buccal mucosa and retromolar trigone.

b: The UICC stage after primary surgical treatment and pathological examination.

Abbreviation: UICC, Union for International Cancer Control.

c: Both the patients diagnosed with lymph node metastases from the primary surgical treatment and patients with isolated lymph node recurrences were considered N+.

d: Tobacco consumption was defined as high if the patient reported a history of >10 pack-years and as low if it was ≤ 10 pack-years.

### Digital image analysis of tumor budding

**Figure 1:**
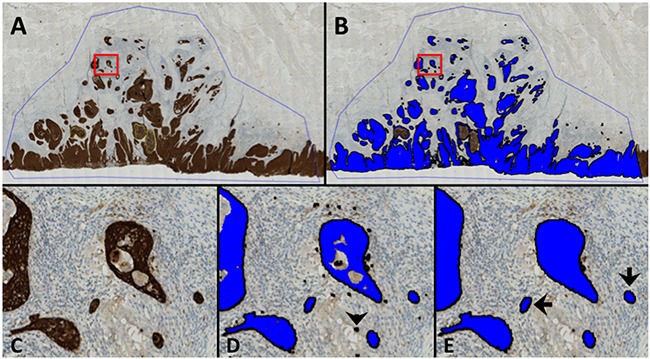
Digital image analysis An example of the digital image analysis. All brown colors, i.e. positively stained areas, were identified and covered with a unique label (blue). The area of each classified tumor island (blue label) was subsequently calculated. If the size of an individual tumor island was less than 950 μm^2^ they were counted as tumor buds, i.e. the Digital Tumor Bud Count (DTBC). **A**. An overview of the scanned tumor slide showing the region of interest (ROI, blue line). The yellow lines in the center of the tumor are necrotic areas, which were marked with another ROI and not analyzed by the software. **B**. As in (A), post image analysis illustrating the classified image where the blue area corresponds to the identified tumor area. **C, D** and **E**. represent the enlarged area of the red box in A and B. D: labeled areas after classification of the tumor tissue by the pre-adjusted threshold. Black arrowhead indicates a minor staining artefact that was unlabeled (see E) since it was < 150 μm^2^. E: Black arrows: Examples of tumor buds with a surface area of < 950μm^2^ (890 μm^2^ and 885μm^2^ for the left and right, respectively) surrounded by a reactive stroma. The middle tumor island in (E) was 1297 μm^2^ and was therefore not counted as a tumor bud.

**Figure 2:**
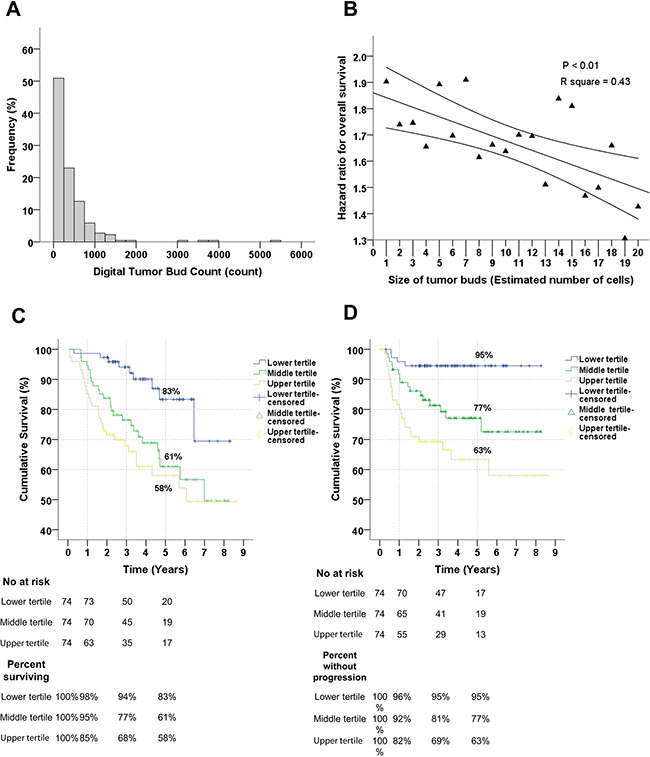
The frequency and survival analyses of the digital tumor bud count **A**. Frequency of tumor buds in patients with OSCC. Note that the majority has between 0 and 1000 buds per section. **B**. Relationship between the size of tumor bud area (i.e. estimated number of cell per tumor island) and the relationship with overall survival per tertile increment. There is a significant linear relationship between the size of the tumor buds and prognostic importance; as the islands of the tumor buds increases its importance in predicting survival diminishes significantly. **C**. Relationship between the Digital Tumor Bud Count (DTBC) and overall survival. The DTBC has been divided into upper, middle and lower tertiles, and the comparison is significant (P < 0.01). **D**. Relationship between the DTBC, divided into tertiles, and progression-free survival, and the comparison is significant (P < 0.01). Notice that almost none of the patients with a DTBC in the lower tertile have a progression after 5 years; 95% are without progression. In C and D: the numbers of patients at risk are shown at the time of 0, 1, 3, and 5 years.

Using digital image analysis, we obtained an objective measure of the degree of tumor dissociation, i.e. a tumor bud count (Figure 1). The total number of tumor buds per tumor section varied considerably from patient to patient (Figure 2A).

### Evaluation of the optimal number of cells in a tumor bud to use to maximize prognostic ability

We evaluated how the prognostic ability of the DTBC changed when tumor buds were defined as areas with between 1 and 20 cells (Figure 2B). We observed that the prognostic value of the DTBC decreased linearly as the tumor island area increased (Figure 2B and Supplementary Figure 2), suggesting that only small tumor islands have prognostic value. Use of a cut-off of 950 μm^2^, which corresponds to fewer than six tumor cells, was a reasonable compromise between predictive ability, significance, and robustness; we therefore proceeded to use this cut-off value in the subsequent analyses (Figure 2B and Supplementary Figure 2). All steps of the analysis were performed blinded.

### Relationship between the digital tumor bud count and survival

We observed that the DTBC, which was divided into tertiles for illustrative purposes, was a significant predictor for OS (Figure 2C) and PFS (Figure 2D, Table 2). Of the prognostic factors that we tested, the DTBC was the best for identifying patients with a poor OS and PFS (Table 2). The DTBC was also a strong predictor of poor survival when used as a continuous variable (data not shown), which suggests a dose-response type relationship between the DTBC and poorer survival.

**Table 2:** Univariate analysis of pathological characteristics impact on overall and progression-free survival

Variables	Overall survival			Progression-free survival		
	Events	HR (95% CI)	*P*	Events	HR (95% CI)	*P*
DTBC	64			41		
*Lower tertile*	9	1		4	1	
*Intermediate tertile*	26	3.0 (1.4-6.5)	0.004	15	4.0 (1.3-12.1)	0.01
*Upper tertile*	29	4.0 (1.9-8.4)	<0.001	22	7.1 (2.4-20.5)	<0.001
Lymph node metastases^a^	67			49		
*None*	36	1		25	1	
*Micrometastases and ITC*	7	1.7 (0.7-3.8)	0.2	6	2.0 (0.8-4.8)	0.1
*Macrometastases*	24	3.8 (2.2-6.4)	<0.001	15	3.1 (1.6-5.8)	0.001
Differentiation grade	66			45		
*Well*	14	1		8	1	
*Moderate*	39	1.6 (0.9-3.0)	0.1	27	2.1 (1.3-3.6)	0.004
*Poor*	13	2.1 (1.0-4.5)	0.06	10	2.7 (1.5-4.9)	0.001
Absolute invasive depth	65	1.1 (1.0-1.2)	0.004	46	1.1 (1.0-1.2)	0.008
Invasive depth (>4 mm vs. <4 mm)	65	1.6 (1.0-2.5)	0.08	46	1.6 (0.9-3.0)	0.09
Stage (cT2 vs. cT1)	67	1.7 (1.0-2.7)	0.04	46	2.5 (1.4-4.4)	0.002
Tumor invasive front(Non-cohesive vs cohesive)	64	1.8 (1.1-3.0)	0.03	43	2.1 (1.1-4.2)	0.03
Perineural invasion (yes vs. no)	64	1.7 (1.0-2.9)	0.05	43	2.2 (1.2-4.0)	0.01
Tumor location	67			46		
*Floor of the mouth*	28	1		22	1	
*Tongue*	28	1.2 (0.7-2.0)	0.5	17	0.9 (0.5-1.7)	0.7
*Other sub-sites*	11	1.9 (0.9-3.8)	0.07	7	1.6 (0.7-3.8)	0.3
Age (per 1 year increment)	67	1.0 (1.0-1.1)	0.005	46	1.0 (1.0-1.0)	0.7
Smoking^b^ (high vs. low)	58	1.5 (0.8-3.1)	0.2	41	0.8 (0.4-1.7)	0.6
Gender (male vs. female)	67	1.0 (0.6-1.6)	1	46	1.5 (0.8-2.6)	0.2

a: The size of the lymph node metastases found during primary surgical treatment.

b: Tobacco consumption was defined as high if the patient reported a history of >10 pack-years and as low if it was ≤ 10 pack-years.

Abbreviations: DTBC, Digital Tumor Bud Count, HR, hazard ratio, CI, confidence interval, ITC, isolated tumor cells.

To test whether DTBC was independently related to OS or PFS, we performed a multivariate Cox regression analysis with conditional forward elimination. We observed that the DTBC was a better predictor of overall survival than the other variables from Table 2, which included absolute invasive depth and lymph node metastases. In fact, only DTBC, lymph node metastases, and age at diagnosis were independent factors for OS (Table 3). We performed correlation analyses to better characterize why only these specific variables were independently related to survival (Supplementary Figure 1).

**Table 3:** Independent factors from the multivariate Cox regression analyses

Multivariate Cox regression	HR (95% CI)	P
**Overall survival**		
DTBC (per tertile increase)	1.6 (1.1-2.2)	0.01
Lymph node metastases^a^	1.7 (1.3-2.2)	<0.001
Age at diagnosis (per 1 year increment)	1.0 (1.0-1.1)	0.007
**Progression-free survival**		
DTBC (per tertile increase)	2.3 (1.5-3.8)	<0.001
Lymph node metastases^b^	1.5 (1.1-2.2)	0.01

a: The hazard ratio for lymph node metastases represents an increase in size from no metastases to macrometastases as seen in Table 2.

Abbreviations: HR, hazard ratio, DTBC, Digital tumor bud count.

### Construction of a clinically relevant model for predicting lymph node metastases

**Figure 3:**
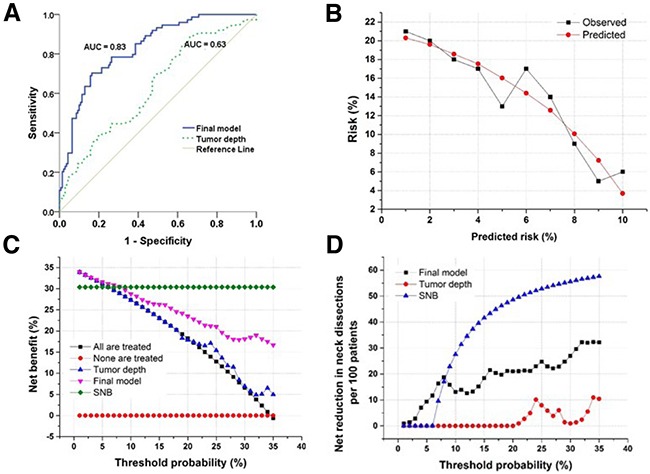
Evaluation of the predictive model and decision curve analysis **A**. Receiver operating curve demonstrating the difference in discriminating between patients with and without occult lymph node metastases based on the final predictive model or tumor depth. The final model is significantly better at discrimination than using tumor invasive depth as a marker. **B**. Calibration plot of the final model showing good agreement between observed and predicted probabilities (P = 0.4, Hosmer-Lemeshow goodness-of-fit). **C**. Decision curve analysis demonstrating the net benefit associated with performing neck dissection based on the markers listed in the figure. Threshold probability is the specific probability of having occult lymph node metastases at which a clinician would choose to perform a neck dissection. The highest curve at any given threshold is the optimal decision-making strategy to maximize net benefit. **D**. Decision curve analysis demonstrating the net reduction in performing neck dissections based on the markers listed in the figure. In the range of relevant threshold probabilities the final model leads to a large reduction in unnecessary neck dissections compared to using tumor depth to evaluate presence of lymph node metastases.

None of the variables in Table 2 was, on its own, a strong predictor of lymph node metastases (data not shown). We therefore constructed a multivariate model to predict lymph node metastases using variables from Table 2 (Supplementary Materials and Methods). The multivariate model was able to predict lymph node metastases with an area under the curve (AUC) of 0.83 (95% CI: 0.78–0.89, *P* <0.001, Figure 3A and variables are shown Supplementary Table 1) compared to an AUC of 0.63 (95% CI: 0.55–0.71, *P*=0.001) using invasive tumor depth as a continuous variable (Figure 3A). To evaluate the robustness of the multivariate model, we performed 10-fold cross-validation of the model and found a cross-validated AUC of 0.79 (95% CI: 0.73–0.85) (Supplementary Materials and Methods).

To better translate the findings from the model to a clinically meaningful decision tool, we performed decision curve analyses and included known clinical parameters, such as absolute invasive depth, for comparison (Figure 3). The decision curve analysis allows a clinician to evaluate the net benefit of different variables based on a threshold probability of having lymph node metastases compared to no intervention or neck dissection (Figure 3C). This analysis demonstrated that if a clinician performed a neck dissection based on an 8% probability of having lymph node metastases, the net benefit of the final model would be markedly better than relying on absolute invasive depth (Figure 3). In addition, at a 10% probability threshold, the model would result in a net benefit comparable to that of performing SNB (Figure 3). In other words, if use of the model is compared to no neck dissection for anyone, then performing neck dissection on the basis of the model is the equivalent of a strategy that finds 27 occult lymph node metastases per hundred patients without conducting any unnecessary neck dissections. Another way of looking at the decision curve analysis is to examine the number of unnecessary neck dissections that would be avoided if the model were used compared to the option of treating all patients with neck dissections. At a threshold probability of 10%, the net reduction in unnecessary neck dissections per 100 patients is 13 using the final model compared to 0 using tumor depth without missing any lymph node metastases.

## DISCUSSION

This study had three major findings. First, it demonstrated that it is possible to obtain a clinically useful DTBC using a simple, semi-automatic, digital image analysis of AE1/AE3 cytokeratin-stained sections, which helps facilitate the translation of the DTBC to clinical practice. Second, it demonstrated that the DTBC is the most powerful prognostic factor in patients with early-stage oral cancer; in fact, this parameter outperformed known clinical risk factors, such as the presence of lymph node metastases and absolute invasive tumor depth. Third, this study demonstrated the development and use of a clinically relevant risk model for lymph node metastasis that incorporates the tumor bud count. Most notably, this model has a markedly higher net benefit than absolute invasive depth, and its use would lead to fewer OSCC patients with a cN0 neck receiving unnecessary neck dissections.

Even though a high tumor bud count has been recognized as an important adverse prognostic factor, primarily in colorectal cancer [21] but also in several other cancer types including OSCC [17, 22–24], tumor budding is not a routine part of pathology reports. We suggest that the major reasons for this lack of clinical translation are 1) the many different tumor budding scoring methods [25]; and 2) the time-consuming nature of counting tumor buds routinely in a pathology department. Tumor bud scoring varies considerably both in terms of which stain is used and in terms of the cut-off value that is used to determine whether the tumor bud count is high [13, 14, 24, 26]. Furthermore, the location of the buds (intratumoral budding [27] vs. budding at the invasive front [28]) and the area that is scored (one high-power field [14] or ten high-power fields [29]) have varied as well, leading to a lack of consensus about the best method(s) to use.

We chose to use a cut-off of an area corresponding to five cells to define a tumor bud in this study. Although this cut-off is somewhat arbitrary, since no studies have evaluated whether other cut-offs would result in better predictive ability, this has been the most consistently used cut-off in the literature. However, we demonstrated that the predictive ability of the tumor bud count decreased linearly as the size of tumor buds decreased. In addition, we showed that the 5-cell cut-off was reasonable when analyzing the impact on OS.

Our proposed DTBC is relatively easy to use, and it has clinical relevance for both prognosis and for the ability to predict lymph node metastases. We thus propose prospectively validating our scoring method, which could aid in a standardization of the tumor bud count method. The implementation of a new, important risk factor is particularly relevant in OSCC, where no clinically useful risk factors have been implemented into clinical practice in recent years. Even more sophisticated pathological scoring methods than the DTBC have been advocated, such as a phosphohistone H3 and KI67 scoring system that are proposed for use in breast cancer [30] and melanoma [31]. Our proposed scoring method makes it possible to perform all of the analyses using the software, except for the initial manual delineation of the tumor area. Therefore, the pathologist's labor needed to obtain a DTBC is minor compared to the quantitative and standardized outcome. We acknowledge that the pathologist would need to identify the tumor block containing the deepest invasion as well as manually drawing a region of interest in the software in order to run the automated algorithm. This should however be compared to the labor and expense involved in step-serial sectioning of sentinel nodes currently performed in many centers. We also acknowledge that the proposed biomarker would only be possible to implement in a fully equipped digital pathology lab, which is however becoming more common across centers.

At many centers, the decision to perform neck dissection is currently based upon SNB results [32]. A recent large randomized study demonstrated better survival in patients that are treated with an upfront neck dissection as opposed to treating neck recurrence [5], which underlines the importance of correct staging of the neck at diagnosis. However, it is well known that a treatment algorithm that offers all patients an upfront neck dissection will lead to overtreatment of approximately 70% of patients, i.e. will have a low net benefit. Since it is not possible to perform SNB in all head and neck cancer centers, other risk factors for harboring lymph node metastases have been proposed that are based on both clinicopathological characteristics and on gene expression studies [6]. The absolute invasive depth of the tumor is one of the most widely used risk factors in clinical use for helping decide whether a neck dissection should be performed [33]. It has thus been suggested that a depth greater than 4 mm should be used as a guide for choosing whether to perform a neck dissection [18, 34, 35]. We demonstrated that the net benefit is lower when using absolute tumor depth (at a cut-off of 4 mm) to predict lymph node metastases versus using our proposed model, which incorporates the tumor bud count. In fact, the benefit of using the DTBC model may be similar to the benefit of using SNB at a low threshold probability of having lymph node metastases, but it requires a two-step procedure. It is also important to acknowledge that even SNB, apart from being both labor intensive and expensive, is not 100% accurate in predicting lymph node metastases [36]. A limitation of our study is that the DTBC needs to be performed on immunohistochemically stained surgical resection specimens, as it is not possible for the software to perform the analysis on standard H&E stained sections. The use of immunohistochemical staining in oral resection specimens is currently not routinely performed on oral cancer specimens. Additionally, it would not be possible to identify patients with a high risk of having lymph node metastases until after the primary surgery, leading to a two-stage procedure. This is however also the case with the current SNB technique.

The decision to offer adjunctive radiotherapy to patients with early-stage oral cancer is currently based primarily upon whether free margins are attained and whether there is extracapsular spread in any lymph node metastasis [3, 4]. It is therefore interesting that the DTBC was the most important predictor of poor survival in this cohort of patients with early-stage oral cancer who are generally considered to have overall good survival. The relevance of the tumor bud count in predicting survival was even better than the presence of lymph node metastases, which is currently considered the most important risk factor for survival in early-stage oral cancer and which is known to decrease survival by up to 50% [37, 38]. Therefore, identification of a valuable new risk marker in early-stage oral cancer could be relevant in deciding which patients with an expected poor prognosis should be offered adjunctive radiotherapy in the future. This needs to be studied in a controlled trial.

In conclusion, this study demonstrated the feasibility of using digital image analysis to obtain a digital tumor bud count from cytokeratin-stained sections from patients with early-stage oral cancer. This has important prognostic implications as the method could be used as a guide for choosing whether to perform neck dissection.

## MATERIALS AND METHODS

### Patients

We used the Reporting Recommendation for Tumor Marker (REMARK) guidelines to conduct and report this study [39]. This cohort has been described previously and includes 253 consecutive treatment-naïve patients with cT1–T2N0 OSCC who were treated at our center from April 2007 to December 2013 [3]. All patients were treated with curative intent and underwent SNB. Histological diagnosis of each tumor was based on examination of a hematoxylin and eosin (H&E) stained tissue section by a consultant head and neck pathologist in accordance with the WHO classification of tumors [40]. The TNM classification followed the International Union Against Cancer, sixth edition 2002 guidelines [41].

From the original tumor sections from the surgical resection specimen, which were routinely H&E stained, a consultant head and neck pathologist (G.L.) identified the tumor block with the deepest invasion for subsequent immunohistochemistry (IHC). IHC staining broad-spectrum cytokeratin was performed on serial sections (Supplementary Methods). Formalin-fixed paraffin-embedded (FFPE) tumor tissue was available from 222 of the 253 patients (88 %), since the non-available tumor tissues had been used for other scientific studies or no spare unstained tissue were left. This left 222 patients for inclusion in the digital image analysis investigation. The Scientific Ethics Committee of the Capital Region of Denmark and the Data Protection Authority approved this study (ID No. H-1-2014-H53).

### Scanning and digital image analysis

The cytokeratin-stained slides were scanned using the Axio Scan Z1 (Carl Zeiss A/S, Birkeroed, Denmark) at 20x magnification; the same standard scanning protocol was used for all samples, and images were subsequently checked manually to ensure good quality images. Slides were rescanned if necessary to ensure high image quality. The digital images were analyzed using Visiopharm® image analysis software (Visiopharm A/S, Hoersholm, Denmark) using a dedicated image analysis module. Since the anti-cytokeratin antibody also stained salivary glands, it was necessary to manually delineate the tumor as a region of interest (ROI; performed by N.J.P. and D.H.J) in order to exclude salivary glands and necrotic tumor areas that could be misinterpreted by the software. The software identified areas that were positively stained for cytokeratin by setting a minimum threshold of differences in contrast for the brown signal (3,3’-diaminobenzidine, DAB) at a level at which the stained areas were clearly distinct from the non-stained areas. This part of the analysis was performed using 5x magnification and the same protocol was applied to all scans. Each independent stained area in the ROI was subsequently marked with a unique digital label by the Visopharm software (Figure 1). Labeled areas less than 150μm^2^ were unlabeled by the software in order to avoid artefacts. Each digitally labeled area was subsequently quantified automatically in μm^2^ by the software, and this information recorded and stored separately for each label. These labels, which represented cross-sectioned tumor tissue, were defined as tumor buds when their area was below a certain size, and the total number of these labels were counted per slide and was considered to be the DTBC (Figure 1 and Supplementary Materials and Methods).

For translational purposes, we estimated the number of tumor cells in separate tumor buds by defining the average cross-section area of a single OSCC cell in a histological slide to be 190 μm^2^. As tumor buds are defined as tumor islands with up to 5 cells, the tumor bud count was subsequently calculated by counting all labels with a size of up to 5 × 190 μm^2^ = 950 μm^2^; the number of unique areas under 950 μm^2^ defined the DTBC. See Supplementary Materials and Methods for further details.

### Statistical analysis

Statistical analyses were performed using SPSS version 22 (SPSS Inc., Chicago, IL, USA) and R statistics version 3.0.3 [42, 43]. The endpoints in the survival analyses were overall survival (OS) and progression-free survival (PFS) [44]. Recurrence at T-site was defined as a new OSCC tumor within 2 cm of the primary OSCC tumor and histological verified within three years since the primary diagnosis, as previously described [45]. *P*-value <0.05 was considered significant. The detailed methods used for the statistical analyses are described in the Supplementary Materials and Methods.

## SUPPLEMENTARY MATERIALS FIGURES AND TABLES


