# Supplementary material for: Construction of a pathological risk model of occult lymph node metastases for prognostication by semi-automated image analysis of tumor budding in early-stage oral squamous cell carcinoma
Source: Oncotarget. 2017 Feb 14;8(11):18227–37. doi: 10.18632/oncotarget.15314 (PMC5392322; doi:10.18632/oncotarget.15314)
Supplement: Supplementary file 1 [file oncotarget-08-18227-s001.pdf]

Construction of a pathological risk model of occult lymph node metastases for prognostication by semi-automated image analysis of tumor budding in early-stage oral squamous cell carcinoma

SUPPLEMENTARY MATERIALS AND METHODS

Immunohistochemistry

The fresh frozen parafin embedded (FFPE) sections (3 μm) were stained on a Ventana Benchmark ULTRA (Roche, Denmark). Antibody concentrations were established by titration. As an internal control for the monoclonal antibodies that were used on serial sections, we evaluated the negatively-stained areas of one slide with negatively-stained areas for reactivity with other antibodies of the same isotype [1]. Supplementary Table 2 describes the staining protocol, antibody, and their previous validation.

Digital Tumor Bud Count (DTBC)

The current definition of tumor budding states that tumor buds should be measured on a single slide as clusters of up to five tumor cells. To evaluate the optimal cut-off of DTBC areas, we calculated the prognostic impact of each specific area of the tumor buds, to illustrate which tumor bud area that, per tertile contributed the most to the Hazard ratio for overall survival (OS). This was performed by analyzing the tumor bud count of all the tumor areas up to 3800um^2 divided up in intervals of 190um^2. A cut off around 1000um^2 was most likely to be optimal, since when adding bigger tumor areas, the prognostic value of tumor buds decreased and the contribution by adding larger tumor bud areas faded (Figure 2B and Supplementary Figure 2).

Statistical analyses

To test for correlations between clinical variables and the DTBC we used Spearmans' rank correlation. We defined (OS) as time from the histological diagnosis of malignancy to death due to any cause. The progression-free survival (PFS) was defined as time from the histological diagnosis of malignancy to time of recurrence at any site. Only histological or cytological verified recurrences were accounted as a true recurrence of the OSCC. The date of the last follow-up was the 6th of October 2015 or the day of death. Alive patients were censored at last follow-up date. Hazard ratios were calculated by univariate Cox regression with log rank tests for each parameter (Table 2). We used Kaplan-Meier curves to illustrate survival differences, with log-rank tests to test for significant differences in survival (Figure 2). Missing data were left out of the analyses (see Table 2 for details).

To evaluate whether the significant variables from the univariate analyses contributed independently to survival we constructed a multivariate Cox regression analysis for OS and PFS. These multivariate models were constructed by inserting all significant variables from the univariate analyses (Table 2), and subsequently use conditional forwards elimination producing a model of only significant factors (Table 3).

To construct a predictive model for lymph node metastases we first computed a bud count per patient. In order to better capture the different possibilities of choosing a cut off to define tumor buds, we computed a bud count per tumor island area from islands of size 1 up to size 20. We therefore ended up with 20 different bud counts per patients, based upon where the cut off of tumor islands was set, from tumor islands with 1 cell (190μm^2) up to tumor islands with 20 cells (3800μm^2, Supplementary Figure 2). Since the number of tumor buds, to some degree correlated to tumor area (and therefore clinical tumor T-stage), these 20 different bud counts per patients were divided by the total histological tumor slide area, leaving another 20 variables (Tumor bud density).

We subsequently performed univariate binary logistic regression and evaluated these variables as well as the clinical variables from Table 2 as predictors for presence of lymph node metastases. Patients were defined as having lymph node metastases when either the SNB demonstrated metastases or the patients experienced isolated lymph node metastases recurrence (6%) during follow-up. Notably, the latter represents failure of the SNB technique. The significant variables was subsequently used in a multivariate binary logistic regression with conditional backwards elimination of the least significant factors until only significant factors remained to construct a model for lymph node metastases (Supplementary Table 1). Predicted probabilities from this model were used to construct receiver-operating curves (ROCs). The validity/discrimination of the ROC curves were tested with c-statistics, where a value of 1.0 indicates perfect discriminatory ability and 0.5 indicates no predictive ability. Calibration, that is, whether the predicted probabilities differed from the observed, was tested with the Hosmer-Lemeshow goodness-of-fit test. A p-value of above 0.05, indicating no significant difference between the observed and the predicted probabilities, is indicative of a satisfactorily

goodness-of-fit. The observed and predicted probabilities were plotted, to graphically represent the calibration.

To evaluate whether the constructed model was over fitted and internally validate the multivariate binary logistic regression model, we performed 10 fold cross validation with the R package 'cvAUC' (version 1.1.0), with all the variables from the previous model as input.

Decision curve analysis

To better translate the findings from the predictive model into clinical useful information we performed decision curve analyses [2] in R statistics with the publicly available code [3]. These curves were constructed with predicted probabilities from the multivariate binary logistic regression model.

Decision curve analysis (DCA) is a method that compares diagnostic tests in order to maximize clinical utility. One of the key measures from a DCA is the threshold probability. In this study the threshold probability is the threshold probability of having lymph node metastases. For example a clinician could think that an optimal threshold probability for performing neck dissection is when there is a 20% chance of harboring occult lymph node metastases, i.e. a threshold probability of 20%. Another clinician might have different threshold probabilities based on the specific patient and clinical situation. The DCA curves allow the clinician to optimize the net benefit of when to choose a neck dissection in the individual situation. It is known that if all patients with a cN0 neck were offered a neck dissection (referred to as "treat all" in this instance), 70% would experience unnecessary morbidity as only around 30% are expected to have occult lymph node metastases ("false negatives"). On the other hand if none were treated with a neck dissection ("treat none") around 70% would receive adequate treatment ("true negatives"). The decision curve analysis allows a clinician to evaluate novel biomarkers for lymph node metastases in a curve where the net benefit of each scenario can be evaluated.

In this study, the DCA was used to compare tumor depth, our multivariate model and the SNB technique as a decision tool in choosing which patient should be offered a therapeutic neck dissection. In other words, to investigate which of these tests would result in the largest net benefit, based on different threshold probabilities.

In this way, for example in this study, at a threshold probability of 8%, the net benefit of using the model was approximately 31%. This means that for every 100 patients who are evaluated using this model compared to a treat no one strategy, 31 patients would be treated appropriately with a therapeutic neck dissection. At the same threshold probability of 8%, the net benefit was approximately 29 % when using tumor depth. Accordingly, at threshold probability of 8% the net benefit of using the final model the net benefit of using the final model versus tumor depth is 31-29, or 2 additional patients would receive appropriate therapy. However the harm associated with neck dissection should also be weighed against the number of patients receiving inappropriate neck dissection. At a threshold probability of 8% the number of patients avoiding neck dissection was approximately 19 per 100 patients with the final model, whereas the number of patients avoiding unnecessary neck dissections were 0 per 100 patients.

REFERENCES

1. Rasmussen OF. Controls. Immunohistochemical staining methods, 5th edn Dako North America, Carpinteria. 2009; 127.
2. Vickers AJ and Elkin EB. Decision curve analysis: a novel method for evaluating prediction models. Medical decision making. 2006; 26:565-574.
3. Vickers AJ. (2016). Statistical Code for Running Decision Curve Analysis - R code. www.mskcc.org/).
4. Tseng SC, Jarvinen MJ, Nelson WG, Huang J-W, Woodcock-Mitchell J and Sun T-T. Correlation of specific keratins with different types of epithelial differentiation: monoclonal antibody studies. Cell. 1982; 30:361-372.
5. Woodcock-Mitchell J, Eichner R, Nelson WG and Sun T-T. Immunolocalization of keratin polypeptides in human epidermis using monoclonal antibodies. The Journal of cell biology. 1982; 95:580-588.

SUPPLEMENTARY FIGURES AND TABLES

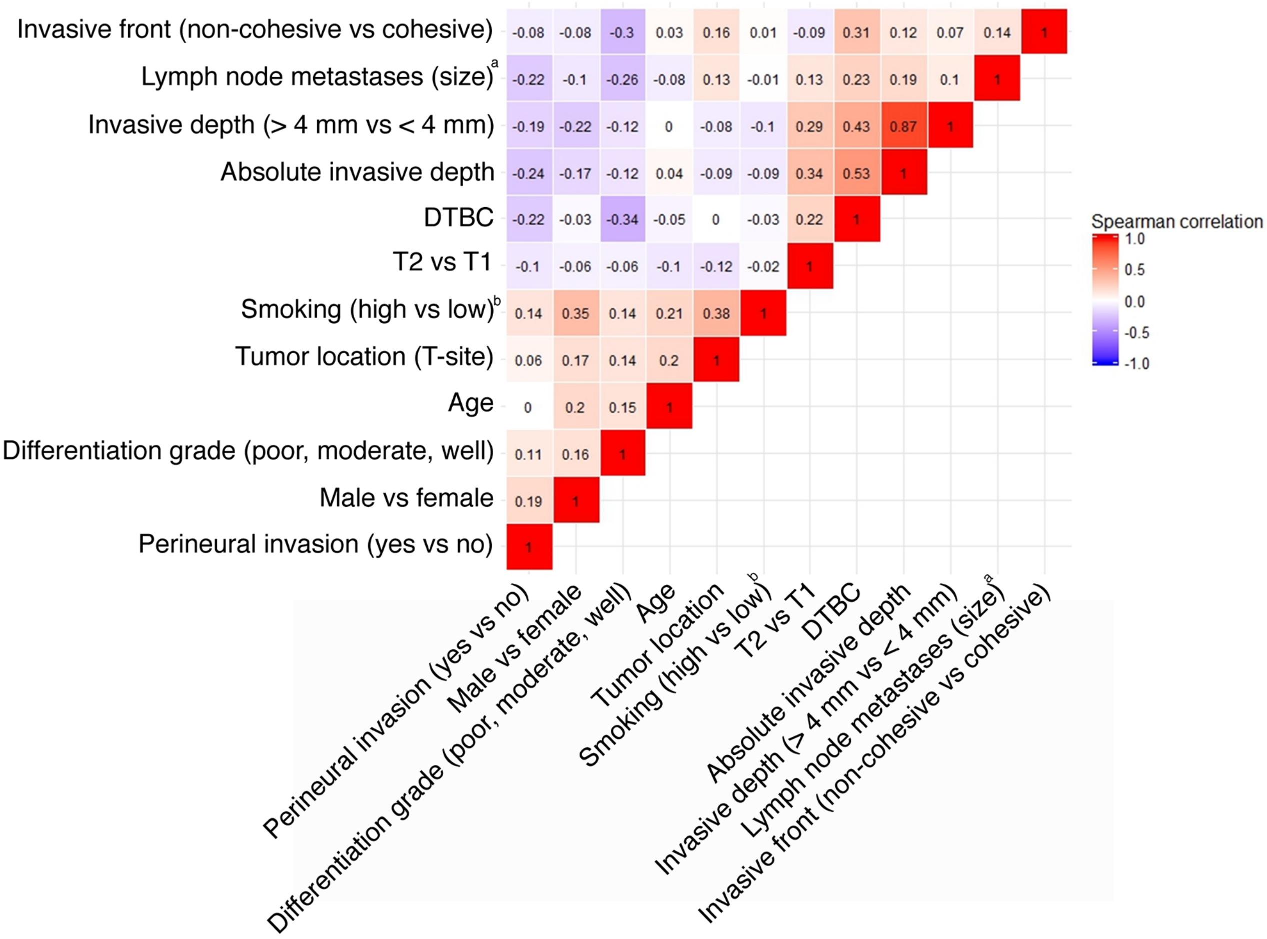

Supplementary Figure 1: Spearman's correlation between Digital Tumor Bud Count and clinicopathological factors. **a.** Isolated tumor cells and micrometastases versus macrometastases. **b.** Tobacco consumption was defined as high if the patient reported a history of >10 pack-years and as low if it was ≤ 10 pack-years. Abbreviation: DTBC, Digital tumor bud count, T2 and T1, clinical T-stage.

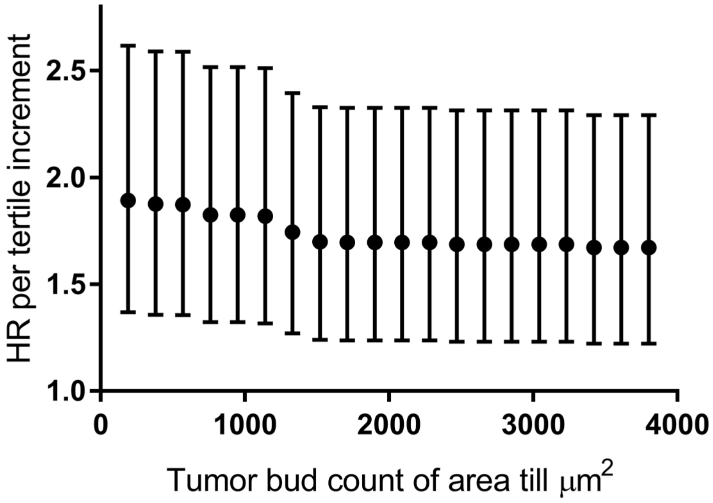

Supplementary Figure 2: The tumor bud size and its prognostic impact. Hazards ratio of overall survival per tertile increment for the cumulative tumor buds count of tumor islands up till the given area. Note that the prognostic impact outlines, as the more of the bigger tumor buds are included in the digital tumor bud count.

Supplementary Table 1: Factor included in the final multivariate logistic regression model

	Exp (B)	Wald	P
Tumor depth[a]	1.23	8.91	0.003
DTBC	1.14	13.5	<0.0001
Differentiation	0.27	14.6	<0.0001
Tumor site[b]	2.00	6.34	0.01
Bud count[I] 1,140um^2	0.73	18.7	<0.0001
Bud count[I] 1,330um^2	1.21	20.3	<0.0001
Bud density[I] of 380um^2	1.08	7.12	0.008
Bud density[I] of 1,330um^2	0.21	17.6	<0.0001
Bud density[I] of 2,090um^2	3.59	6.24	0.01
Bud density[I] of 3,040um^2	6.84	8.25	0.004

The cytokeratin anti-body staining protocol and validation. Abbreviation: HIER, Heat-induced antigen retrieval

Supplementary Table 2: Antibodies and staining protocol

Antigen	Clone	Catalogue number	Species	Isotype	Source	Pre-treatment	Dilution	Detection system	References
**Cytokeratin**									
	AE1/ AE3	M3515	Mouse	IgG1	DAKO	HIER in CC1	1:200	Ultraview	[4, 5]

a: This variable was included in the model as continuous variables.
b: The floor of the mouth versus oral part of the tongue and the other sub sites of the oral cavity. Bud density was calculated by dividing the count of the specific area with the total tumor area. For further description of the bud density and bud count for the areas chosen, please see Supplementary Materials and Methods.
Abbreviation: DTBC, Digital tumor bud count.
